# Valorizing Tree-Nutshell Particles as Delivery Vehicles for a Natural Herbicide

**DOI:** 10.3390/mps7010001

**Published:** 2023-12-20

**Authors:** Jong H. Kim, Kathleen L. Chan, William M. Hart-Cooper, DeAngela Ford, Kaydren Orcutt, Jeffrey D. Palumbo, Christina C. Tam, William J. Orts

**Affiliations:** 1 Foodborne Toxin Detection and Prevention Research Unit, Western Regional Research Center, Agricultural Research Service, United States Department of Agriculture, 800 Buchanan St., Albany, CA 94710, USA; kathy.chan@usda.gov (K.L.C.); deangela.ford@usda.gov (D.F.); jeffrey.palumbo@usda.gov (J.D.P.); christina.tam@usda.gov (C.C.T.); 2 Bioproducts Research Unit, Western Regional Research Center, Agricultural Research Service, United States Department of Agriculture, 800 Buchanan St., Albany, CA 94710, USA; william.hart-cooper@usda.gov (W.M.H.-C.); kaydren.orcutt@usda.gov (K.O.); bill.orts@usda.gov (W.J.O.)

**Keywords:** agricultural by-products, pre-emergent, post-emergent, salicylaldehyde, tree nutshells, valorization, weed control

## Abstract

The United States is a principal producer of tree nuts (almonds, pistachios, and walnuts), resulting in the generation of excess of tree-nutshell by-products each year, with few market outlets. A nutshell is an essential, lignocellulosic layer that protects a kernel (seed) from the environment during cultivation. The objective of this study was to develop nutshell by-products as herbicide delivery systems, which would not only enable sustainable weed control in fields but also increases nutshell value and reduce the cost of waste disposal. We recently identified a natural salicylaldehyde (SA) that emits volatiles with both herbicidal and antifungal properties. In this study, walnut shell particles saturated with 0.8 to 1.6 M SA were developed as delivery vehicles for SA to soil, which allowed for the controlled release of an SA fumigant for weed control. The pre- and post-emergent herbicidal efficacy of SA was investigated using model monocot (*Lolium arundinaceum* (Schreb.) Darbysh; turfgrass) and dicot (*Brassica rapa* var. *pekinensis*; Chinese cabbage) plants. We compared (1) the effects of different types of solvents for dissolving SA (dimethyl sulfoxide (DMSO) and ethanol (60%, *v*/*v*)), and (2) the effect of covering soil with plastic layers (i.e., soil pasteurization) or not covering soil during SA fumigation using nutshells. Results: In the pre-emergent herbicidal testing with the soil covered, the dicot plants exhibited levels of higher susceptibility to SA in DMSO emitted from nutshells when compared to the monocot plants. The seed germination frequencies in the dicots were 15% and 1% with 0.8 and 1.6 M SA, respectively, while those in the monocots were 32% and 18%, respectively, under the same test conditions. In the post-emergent herbicidal testing with the soil covered, the growth of both the monocot and dicot plants was completely prevented after 5 to 7 days of SA fumigation, resulting in the deaths of entire plants. It was noteworthy that in the post-emergent herbicidal testing, SA dissolved in ethanol (60%, *v*/*v*) completely disrupted the growth of the monocot and dicot plants as early as 3 days after SA emission from the nutshells, even without the soil being covered. Tree-nutshell particles could serve as effective SA delivery vehicles with controlled release capabilities for SA. The SA exhibited pre- and post-emergent herbicidal activities against the monocot and dicot plants at most growth stages. SA (0.8 and 1.6 M) dissolved in ethanol (60%, *v*/*v*) might exert a synergism for higher herbicidal activity after emission from nutshells. Since tree nuts capture/store a substantial amount of carbon over their life-cycles, the new and sustainable utility of using nutshells not only reduces carbon emissions but also valorizes tree-nut by-products, thus benefitting the tree-nut industry.

## 1. Introduction

Agricultural industries generate an excessive amount of crop by-products annually, with negative impacts on the environment [1,2,3]. For instance, California, United States, produces approximately 80% of the world’s almonds, resulting in 4.8 billion pounds of hulls, 1.9 billion pounds of nutshells, and 4.3 billion pounds of tree woody biomass per year [4]. Regarding the nutshell by-products, although there have been efforts to reutilize and valorize them within a circular bioeconomy platform, there are few market outlets for the nutshells, except for their use in the dairy industry for animal bedding [4]. At present, whole orchard recycling, namely, the incorporation of by-products back into an orchard’s soil, is an alternative method for managing by-products (which also helps carbon sequestration) [5]. However, as determined in other agricultural by-products, tree-nut by-products such as nutshells are commonly contaminated with environmental pathogens (see Supplementary Figure S1; [6]). Hence, ensuring that the by-product recycling process does not negatively affect soil health by transferring pathogens to fields is critical. Accordingly, there is a high demand for the identification of new and sustainable methods to utilize tree nutshells to reduce mounting stockpiles of accumulated nutshells, which can also alleviate negative impacts on the environment [7,8,9].

Walnut shells are mainly composed of a lignocellulosic material which contains cellulose, hemicellulose, and lignin [10]. Of note, walnut shells, either in their nascent (unprocessed) or processed (e.g., activated charcoal, functional group addition, etc.) forms, have recently been investigated as a potent, plant-based biosorbent to adsorb/eliminate pollutants, such as heavy metals, toxic chemicals, pharmaceuticals, industrial dyes, etc., from fields [7,10]. Conversely, we hypothesized that walnut shells could also function as delivery vehicles for chemicals, such as soil fumigants (pesticides), allowing for the controlled release of molecules necessary for pest control.

We previously identified that the natural product salicylaldehyde (SA) emits volatiles with both antifungal and herbicidal properties [6]. SA is a natural product classified as a generally-recognized-as-safe (GRAS) molecule [11,12]. SA has been utilized as a food additive (flavoring agent) or an intermediate for synthesizing pharmaceuticals. In our previous studies, SA exhibited strong antifungal as well as anti-mycotoxigenic activities [13,14], where fungal mutants deficient in antioxidative defense systems were vulnerable to SA treatment. Therefore, we reasoned that SA disrupts cellular redox homeostasis in fungal pathogens [13,14]. However, thus far, the molecular mechanism for the herbicidal activity of SA has not been studied or characterized. Considering that many phenolic compounds, such as salicylic acid, possess allelopathic activities [15,16], we surmised that SA might also act as an allelochemical in the environment, inhibiting the growth of surrounding plant species.

Since SA exists as a vapor in the atmosphere (its vapor pressure value is 5.93 × 10^−1^ mm Hg at 25 °C), an SA molecule exhibits high mobility in a field, and then it is emitted from the dry soil. Therefore, SA may be applied as a soil fumigant in an orchard [17]. The advantage of SA is that it has been exempted from requiring a tolerance by the United States Environmental Protection Agency (EPA), indicating that it can be used as an inert ingredient in pesticide formulations (to be applied to growing crops and raw agricultural commodities) at concentrations of up to 14% by weight of the pesticide formulation [18].

We previously determined that tree nutshell particles (i.e., walnut shells) can be used as an SA delivery vehicle to a target site for weed control [6]. By covering soils using plastic layers, SA emitted from nutshell vehicles not only prevented the germination of invasive or native weeds but also inhibited the growth of fungal contaminants on the surface of the nutshells or weed seeds, and thus, they could prevent the transferring of pathogens to fields during application. In crop fields, a soil covering comprised of plastic layers is also termed “soil pasteurization (or solarization)”, and this method which uses sunlight to control agricultural pests, such as weeds, microbes, and/or insects, in soil [19,20]. Therefore, the success of soil pasteurization relies upon weather conditions or a geographic location that can ensure the availability of enough solar radiation during the sanitation process. Notably, since SA can function as a potent fumigant molecule, combining the application of SA with a soil covering could enhance the pre-emergent herbicidal efficacy of a system even at much lower temperatures (e.g., room temperature) [6].

In a previous study, we used dimethyl sulfoxide (DMSO) [21] as a solvent to dissolve SA before applying it to soil using walnut shells as a delivery vehicle. In this investigation, process optimization for weed control was investigated by: (1) utilizing ethanol (60%, *v*/*v*) as an alternative solvent to dissolve SA as ethanol has recently been shown to improve the efficacy of other herbicides [22], and hence, we reasoned that the ethanol-dissolved SA would enhance the herbicidal activity of SA, and (2) comparing the herbicidal efficacy of SA between covered and non-covered soil surfaces with plastic layers (namely, with or without soil pasteurization), thus helping to reduce the negative environmental impact of plastic use in agricultural processes.

## 2. Materials and Methods

### 2.1. Chemicals and Plastic Bags

Salicylaldehyde (SA) was purchased from Sigma Aldrich Co. (St. Louis, MO, USA). The SA was dissolved in dimethyl sulfoxide (DMSO; AMRESCO Co., Solon, OH, USA) or 60% (*v*/*v*) ethanol before application. The plastic layers used in this study were: (1) a translucent bag (inner layer): 91 cm × 61 cm, clear polypropylene, 2 mil thickness (0.0508 mm); Fisher Scientific, Pittsburgh, PA, USA, and (2) a dark brown bag (outer layer): 99 cm × 84 cm, linear low-density polyethylene, 1.22 mil thickness (0.030988 mm); Envision Industries, Wichita, KS, USA.

### 2.2. Plant Seeds

*Brassica rapa* var. *pekinensis* seeds (Chinese cabbage; a dicotyledonous (dicot) plant) and grass seeds (a monocotyledonous (monocot) plant), which were mixtures of tall fescue (turfgrass; *Lolium arundinaceum* (Schreb.) Darbysh) including dynamite GLS tall fescue (30%; resistant to *Pythium* blight and grey leaf spot), titanium GLS tall fescue (30%; resistant to grey leaf spot), firecracker GLS tall fescue (30%; resistant to grey leaf spot), and bluegrass (*Poa pratensis* L.; Kentucky bluegrass arrowhead (10%; resistant to leaf spots)) [23], were procured from plant nurseries (Oakland, CA, USA and Albany, CA, USA, respectively). Tall fescue and bluegrass are cool-season, perennial grasses [24] while *B. rapa* var. *pekinensis* is a biennial herbaceous vegetable [25]. The seeds were maintained at room temperature (22 °C) until use.

The dicot seeds (*B. rapa* var. *pekinensis*) were soaked in sterile water overnight before planting, resulting in a 98% germination rate for the seeds (the same germination frequency was observed with no water-soaking). The monocot seeds (*L. arundinaceum* and *P. pratensis* L.) were planted in soil without water-soaking (for optimum dispersal of the seeds during planting), which led to an 82% germination frequency (an 86% germination frequency was achieved with water-soaking).

### 2.3. SA Herbicidal Efficacy in Soil

The pre- and post-emergent herbicidal efficacies of the SA was investigated in soil (garden soil purchased from a local store, Berkeley, CA, USA). Four plastic trays (22 cm × 50 cm × 15 cm) were half-filled with soil, and 1 g of seeds (*B. rapa*: 295 ± 9 seeds; grass: 316 ± 31 seeds) were sown (~0.5 cm deep) in two rows in parallel (first row: *B. rapa* seeds, second row: grass seeds) per tray. In this study, walnut shell particles (6/10 mesh) (Kramer Industries, Inc., Piscataway, NJ, USA) were used as the delivery vehicle for the SA. The walnut shells were saturated with DMSO (control) or SA (0.8 or 1.6 M) overnight before application to the soil trays, as described previously [6].

#### 2.3.1. Pre-Emergent Herbicidal Efficacy of SA (with a Soil Covering and DMSO)

For the pre-emergent herbicidal efficacy of SA with a soil covering (mimicking soil pasteurization), SA dissolved in DMSO was prepared for each tray as: (1) no SA and without nutshells (control), (2) no SA and with nutshells (10 g; received DMSO only) (control), (3) SA 0.8 M and with nutshells (10 g), and (4) SA 1.6 M and with nutshells (10 g). To mimic the soil pasteurization practice, the trays were covered with two plastic layers (outer and inner layers; see 2.1 above), and they were incubated in the dark (22 °C) for seed germination (seed sowing: 12 May 2023). After 7 days of seed sowing, the plastic covers were removed from the trays and the trays were moved to a light (12 h)/dark (12 h) cycle at 22 °C. The germination and growth of the plant seeds were monitored at the time of the plastic-layer removal (19 May 2023), and thus, 7 days elapsed from sowing to monitoring.

#### 2.3.2. Post-Emergent Herbicidal Efficacy of SA (with a Soil Covering and DMSO; Method 1)

For the post-emergent herbicidal efficacy of SA with a soil covering, both the monocot and dicot seeds (1 g each) were sown in four soil trays without SA treatments (12 May 2023), and then each soil tray was covered with two plastic layers (outer and inner layers) and incubated in the dark (22 °C) for seed germination. After 7 days of seed sowing (plant length: ~5 cm), the plastic covers were removed from the trays for the SA treatments (19 May 2023). SA dissolved in DMSO was prepared for each tray as follows: (1) no SA and without nutshells (control), (2) no SA and with nutshells (10 g; received DMSO only) (control), (3) SA 0.8 M and with nutshells (10 g), and (4) SA 1.6 M and with nutshells (10 g). The trays were covered again with two plastic layers and moved to a light (12 h)/dark (12 h) cycle at 22 °C. The post-emergent herbicidal efficacy of SA was monitored after and additional seven-day incubation period (26 May 2023); hence, 14 days elapsed from sowing to monitoring.

#### 2.3.3. Post-Emergent Herbicidal Efficacy of SA (with a Soil Covering and DMSO; Method 2)

The post-emergent herbicidal efficacy of SA was tested further on the mature plants (extended growth) with a soil covering. Both the monocot and dicot seeds (1 g each) were sown in two soil trays without SA treatments (12 May 2023), and then each soil tray was covered with two plastic layers (outer and inner layers) and incubated in the dark (22 °C) for seed germination. After 7 days of seed sowing (plant length: ~5 cm), the plastic covers were removed from the trays (19 May 2023), and both the monocot and dicot plants were grown further under a light (12 h)/dark (12 h) cycle at 22 °C. After 14 days of extended plant growth (2 June 2023), SA dissolved in DMSO was applied to the soil trays as follows: (1) no SA and without nutshells (control), and (2) SA 1.6 M and with nutshells (10 g). The trays were covered again with a single layer of plastic (translucent) and subjected to a light (12 h)/dark (12 h) cycle at 22 °C. The post-emergent herbicidal efficacy of SA was monitored after an additional 5 days of incubation (7 June 2023), and hence, 26 days elapsed from sowing to monitoring.

#### 2.3.4. Post-Emergent Herbicidal Efficacy of SA (without a Soil Covering and with 60% Ethanol (*v*/*v*); Method 3)

The post-emergent herbicidal efficacy of SA was tested on both the monocot and dicot plants without a soil covering (except for the seed germination period). Both the monocot and dicot seeds (1 g each) were sown in four soil trays without SA treatments (10 July 2023), and then each soil tray was covered with two plastic layers (outer and inner layers) and incubated in the dark at 22 °C for seed germination. After 7 days of seed sowing (plant length: ~5 cm), the plastic covers were completely removed from the trays (17 July 2023), and both the monocot and dicot plants were grown further under a light (12 h)/dark (12 h) cycle at 22 °C. After 14 days of extended plant growth (31 July 2023), SA dissolved in 60% ethanol (*v*/*v*) was applied to the soil tray as follows: (1) no SA and without nutshells (control), (2) no SA and with nutshells (20 g; receiving 60% (*v*/*v*) ethanol only) (control), (3) SA 0.8 M and with nutshells (20 g), and (4) SA 1.6 M and with nutshells (20 g). The trays were subjected (without their plastic coverings) to a light (12 h)/dark (12 h) cycle at 22 °C. The post-emergent herbicidal efficacy of SA was monitored for an additional 15 days of incubation (15 August 2023), and hence, 36 days elapsed from sowing to monitoring.

### 2.4. SA Antifungal Efficacy on Tree Nutshells

Almond and pistachio nutshells were obtained from a local tree-nut farm (Valley Orchard, LLC, Fresno, CA, USA). Four to five tree nutshell particles, depending on their sizes, were aseptically placed onto potato dextrose agar (PDA; BD Life Sciences, Franklin Lakes, NJ, USA) and maintained at 28 °C. The level of fungal contamination was monitored for 72 h.

Then, the level of the antifungal activity of SA against fungi naturally contaminated on the surface of tree nutshells (almond, pistachios) was examined on PDA according to the modified method described previously [26]. SA (0.8 or 1.6 M) dissolved in 60% ethanol (*v*/*v*) was applied to membrane filters (2.5 cm diameter; GE Healthcare, Chicago, IL, USA). SA- or ethanol (control) -saturated filters were positioned onto a PDA Petri plate (100 mm × 15 mm; Corning Inc. Life Sciences, Tewksbury, MA, USA). Two to three tree-nutshell particles, depending on their sizes, were aseptically positioned onto the other half of each PDA. The test plates were incubated at 28 °C to determine the susceptibility of the fungal contaminants to the SA. The control plates contained an ethanol (60%, *v*/*v*) filter only. Fungal growth was monitored for 96 h.

### 2.5. Statistical Analysis

Statistical analysis to determine the student’s *t*-test was performed using “Statistics to use” tool [27]. Paired data for the sizes of plants “with or without pre-emergent SA treatments” or “with or without nutshells” were analyzed, where the *p* value < 0.05 was considered significant.

## 3. Results and Discussion

### 3.1. Pre-Emergent Herbicidal Efficacy of SA (w/ Soil Covering, DMSO)

We previously determined SA’s herbicidal activity on various weed species [6], including *Centaurea solstitialis* (dicot) (Yellow starthistle; California invasive weed), *Salsola tragus* (dicot) (Russian thistle; California invasive weed), *Genista monspessulana* (dicot) (French broom; California invasive weed), *Lagurus ovatus* (monocot) (Bunny Tails Grass; ornamental grass), and *Schizachyrium scoparium* (monocot) (Little Bluestem; USA native grass).

In this study, the pre-emergent herbicidal activity of SA was tested against model dicot and monocot plants, namely, *B. rapa* var. *pekinensis* and grass seeds. Worthy of note, many *Brassica* sp. plants including edible crops could become weedy or invasive and prevent the germination of economic plants in fields. Therefore, these *Brassica* sp. are listed as restricted/prohibited noxious weeds in the United States and Canada [28,29]. For example, the edible Indian mustard *B. juncea* is listed as a noxious weed in the Midwestern states of the United States, and hence, they should not be cultivated in areas involving a danger of dissemination [30].

**Figure 1:**
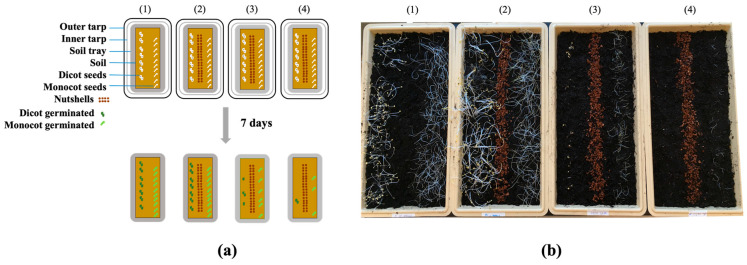
Pre-emergent herbicidal efficacy of SA with a soil covering using plastic layers: (**a**) diagram describing how each soil tray was treated: (1) control (without SA and without nutshells), (2) control (without SA and with nutshells), (3) SA (0.8 M and with nutshells), (4) SA (1.6 M and with nutshells); and (**b**) differential plant growth after the SA treatments described in diagram (**a**).

As shown in Figure 1, SA dissolved in DMSO exerted a potent pre-emergent herbicidal activity against the monocot and dicot plants with soil coverings. For example, we observed a notable reduction in the seed germination frequencies in the dicot plants where the total number of seeds germinated/survived were 43 (15% germination) and 3 (1% germination) after treatment with 0.8 and 1.6 M of SA, respectively.

Although the seed germination frequencies in the monocot plants were also reduced, the total numbers of seeds that germinated/survived were 100 (32% germination) and 56 (18% germination) after treatment with 0.8 and 1.6 M of SA, respectively. These results indicated that *Brassica* (a dicot) was more susceptible to herbicidal treatment with SA compared to grass (a monocot).

**Figure 2:**
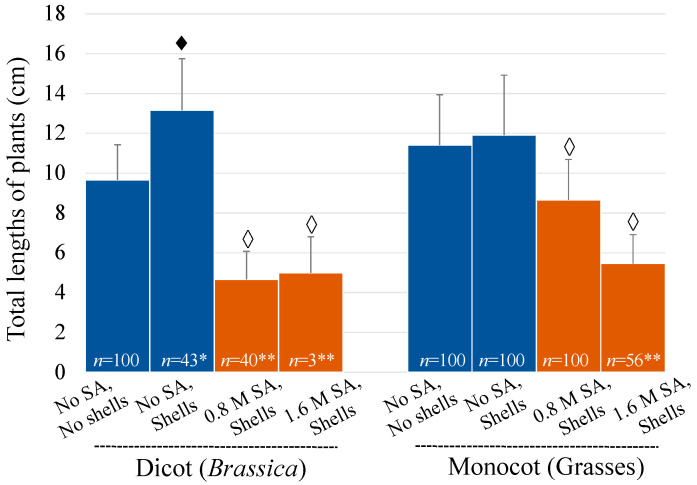
Sizes of the plants with or without the pre-emergent SA treatments. *, number of plants counted (based on no fungal contamination); **, number of plants that survived; ♦, *p* < 0.05 (vs. “no SA, no shells” control); and ◊, *p* < 0.05 (vs. “no SA, shells” control).

In general, the sizes of both the monocot and dicot plants that survived after the SA treatments were also smaller than those without the SA treatments (Figure 2). The germinated plants in the “with nutshell” control were relatively taller (*p* < 0.05 for the dicots) compared to those in the “without nutshell” control. The elucidation of the mechanism of this growth enhancement warrants future investigation. The dicots from the “with nutshell” control (solvent only) were mostly contaminated with environmental fungi during cultivation, and so the forty-three plants not contaminated were selected for examination, as shown in Figure 2.

### 3.2. Post-Emergent Herbicidal Efficacy of SA (w/ Soil Covering, DMSO; Method 1)

The post-emergent herbicidal activity of SA was investigated against *B. rapa* var. *pekinensis* and the grass seeds with soil coverings. The plants were germinated and grown (~5 cm length) for 7 days without SA treatments (without nutshells), and then SA was applied at the respective concentrations (0.8 and 1.6 M dissolved in DMSO and with nutshells).

**Figure 3:**
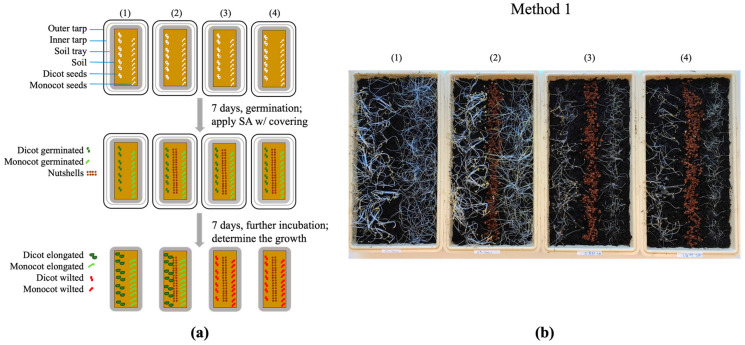
Post-emergent herbicidal efficacy of SA with soil covering using plastic layers: (**a**) diagram describing how each soil tray was treated: (1) control (without SA and without nutshells), (2) control (without SA and with nutshells), (3) SA (0.8 M and with nutshells), and (4) SA (1.6 M and with nutshells); and (**b**) differential plant growth after the SA treatments described in diagram (**a**).

As shown in Figure 3b(3),(4), both the monocot and dicot plants receiving SA ceased development after germination (no further growth), while those incubated without SA continued to grow (Figure 3b(1),(2)), resulting in differences in the plant sizes.

Of note, as observed in the “pre-emergent” testing (above), there was a serious fungal contamination in both “control” trays with soil coverings after 14 days of incubation, resulting in the infection of the entire set of test plants. This infection with the environmental fungi prevented further numerical analysis, i.e., measurements of the differences in the plant sizes. However, the SA-treated trays showed no indication of fungal contamination, indicating that the intrinsic antifungal activity exerted by SA prevented the growth of fungal contaminants (see also Supplementary Figure S1).

Collectively, SA also exhibited post-emergent herbicidal activities against both the monocot and dicot plants in the soil tray assays.

### 3.3. Post-Emergent Herbicidal Efficacy of SA (with a Soil Covering and DMSO; Method 2)

The post-emergent herbicidal activity of SA was investigated against *B. rapa* var. *pekinensis* and the grass seeds after the “prolonged” growth of the plants after germination with soil coverings. The plants were germinated and grown for 21 days without SA treatment (and without nutshells), and then SA dissolved in DMSO was applied at 1.6 M. The plants were then cultivated for an additional 5 days.

**Figure 4:**
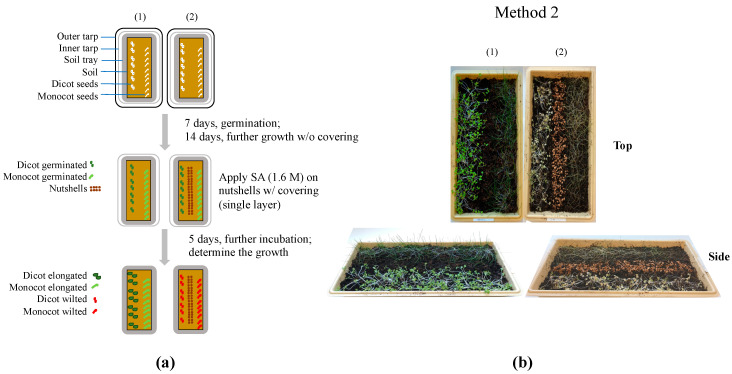
Post-emergent herbicidal efficacy of SA with soil covering using plastic layers: (**a**) diagram describing how each soil tray was treated: (1) control (without SA and without nutshells), and (2) SA (1.6 M and with nutshells); and (**b**) differential plant growth after the SA treatments described in diagram (**a**).

As shown in Figure 4, both the monocot and dicot plants receiving SA exhibited complete wilting (they collapsed), while the plants without SA continued to grow, resulting in differences in the plants’ colors (green vs. brown). Therefore, the post-emergent herbicidal efficacy of SA was determined at both the early (Figure 3) and mature (Figure 4) stages of plant growth, confirming the potent herbicidal activity of SA at various growth stages of plants, including seed germination (pre-emergent; see above).

### 3.4. Post-Emergent Herbicidal Efficacy of SA (without a Soil Covering and with 60% Ethanol (v/v); Method 3)

While soil pasteurization is a sustainable approach for pest control in fields [31], there is growing concern regarding plastic contamination in agricultural soils [32,33,34,35]. Currently, the agricultural application of plastics includes various sectors/stages, such as the nursery stage through to the postharvest crop stage, with the worldwide consumption of plastics by agricultural and agriculture-related industries reaching 2,250,000 tons of plastics per year [35].

Therefore, as proof-of-concept, we also investigated the post-emergent herbicidal activity of SA against *B. rapa* var. *pekinensis* and the grass seeds without soil coverings. The testing was performed to evaluate whether the level of controlled release of the fumigant SA from the nutshell vehicles would be sufficient to effectively control weed growth when plastic covers were not provided.

The plants were germinated and grown for 21 days without SA treatment (and without nutshells), and then SA was applied at the respective concentrations (0.8 and 1.6 M, respectively) dissolved in 60% (*v*/*v*) ethanol. We initially tried to determine the optimum concentration of ethanol to dissolve SA by examining 10 to 100% ethanol, and we found that SA could be dissolved in as low as 60% ethanol. After the SA treatments, the plants were further cultivated for 3 to 15 days.

**Figure 5:**
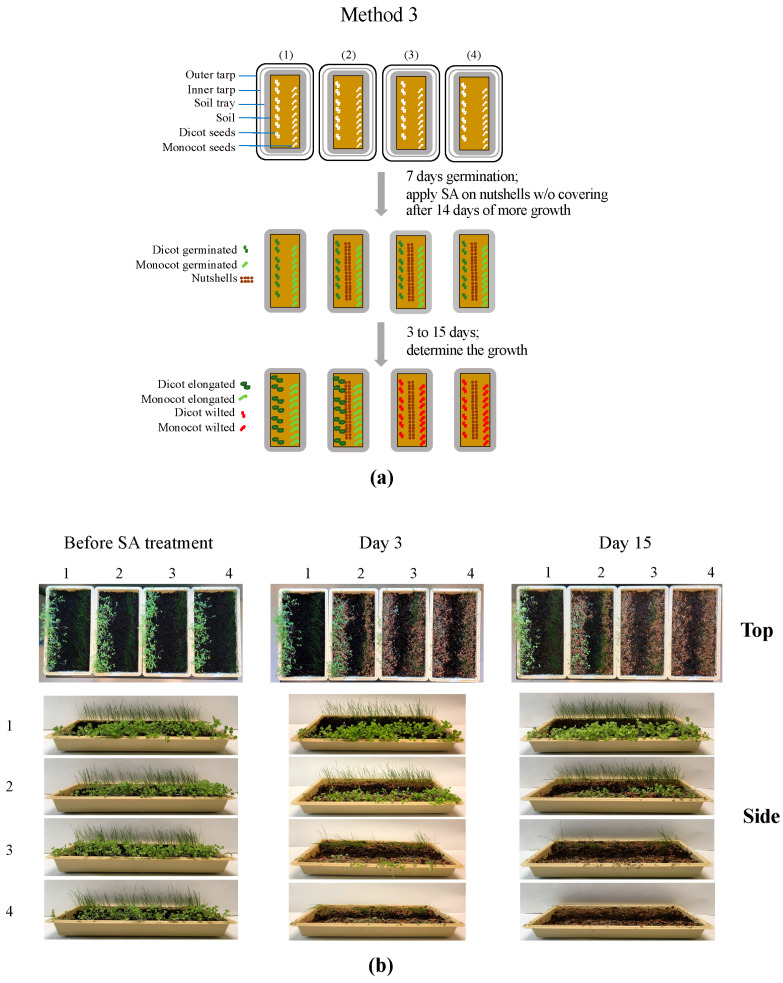
Post-emergent herbicidal efficacy of SA without soil covering using plastic layers: (**a**) diagram describing how each soil tray was treated: (1) control (without SA and without nutshells), (2) control (without SA and with nutshells), (3) SA (0.8 M dissolved in ethanol and with nutshells), and (4) SA (1.6 M dissolved in ethanol and with nutshells); and (**b**) differential plant growth after the SA treatments described in diagram (**a**).

As shown in Figure 5, even without soil coverings, both the monocots and dicots receiving SA could not grow further, resulting in complete plant death (collapsed, with browning) at as early as 3 days after SA treatment compared to the “No SA” controls. Therefore, the post-emergent herbicidal efficacy of SA was also demonstrated at the mature stage of plant growth without soil coverings using SA dissolved in ethanol.

It is noteworthy that we observed a modest decrease in weed growth in the “ethanol control” tray compared to the no treatment control, indicating that the level of ethanol applied may negatively affect plant growth, resulting in a synergism with SA for herbicidal activity (see below). Table 1 summarizes the procedures and methods of SA application used in this investigation and the outcome of each treatment.

In this study, we examined the pre- and post-emergent herbicidal efficacy of SA using monocot and dicot plants, and walnut shells were utilized as the SA delivery vehicle. We compared the types of solvents for dissolving SA and the effect of soil non-covering with plastic layers during SA fumigation. We investigated whether the level of ethanol-dissolved SA emitted from the nutshell vehicle would suffice for the effective control of weeds when plastic layers were not provided. We determined that walnut shell particles could serve as an effective SA delivery vehicle. SA emitted from the walnut shells presented potent pre- and post-emergent herbicidal activities in both the with and without soil covering platforms. SA also exerted herbicidal activities against the monocot and dicot plants at various growth stages (i.e., seed germination and early and mature plant growth after germination).

Weeds are one of the most damaging risks in crop production, and therefore, weed control in agricultural fields is very important for the proper production of crops as well as for ensuring food safety and food security [36,37,38]. Moreover, if not controlled properly, weeds in a field can host harmful pests such as fungi and insects that damage economic crops. Since there are also increasing incidences of herbicide-resistant weeds, it is necessary to develop new and sustainable weed control agents or systems [39].

The mycotoxin-producing fungi *Fusarium* species are good examples for infecting weeds. *Fusarium* species are agricultural pathogens infecting monocot crops. However, *Fusarium* species can also infect various weeds surrounding crop fields, resulting in serious mycotoxin contamination in cultivated crops [40,41,42]. For instance, gramineous weeds surrounding wheat fields can function as alternative hosts for the pathogens *Fusarium graminearum* and *F. asiaticum*, which also produce the mycotoxins 3-acetyldeoxynivalenol and 15-acetyldeoxynivalenol or nivalenol, thus threatening public food safety [40] (Table 2). Similarly, in a study performed in Europe, weeds surrounding spring wheat fields were infected by *F. avenaceum* or *F. graminearum* [41]. These *Fusarium* species also produce several mycotoxins including deoxynivalenol, enniatin A, T-2 toxin (T-2), and moniliformin. Importantly, the study further determined that the type of mycotoxin in the wheat grain depended mainly on the *Fusarium* species infecting the weeds [41]. Of note, the cessation of herbicide applications unexpectedly increased mycotoxin contamination near maximum thresholds in corn fields, which could lead to market restrictions [42]. Altogether, these studies highlighted the significance of proper weed control in fields that can also prevent mycotoxin contamination in economically important crops (Table 2).

From the perspective of the tree-nut industry, concerning by-product management, the “Almond Orchard 2025 Goal” set by the almond industry in California, United States, is to achieve zero agricultural waste by 2025. However, there are almost no market outlets or waste management platforms for nutshells. Therefore, the orchard recycling of tree-nut by-products is currently a compelling method for managing agricultural waste. Considering that tree nuts capture and store a substantial amount of carbon over their life-cycles, the effective re-utilization of their by-products, in addition to orchard recycling, can be one of the key components to reducing carbon emissions as well. It is noteworthy that California almond-bearing acreages are continually increasing (2022: 1,370,000 Acres) [43], and hence, the generation of increased amounts of tree-nut by-products is inevitable in the future. Therefore, finding effective ways to manage agricultural by-products is a highly important and timely endeavor. In California, approximately 9% of the total tree-nut cultivation expenses are spent on the control of weeds, such as the widespread alkaliweed (*Cressa truxilensis*), among others [44]. Accordingly, the SA weed control system investigated in the current study will contribute to cost-effective weed management in tree-nut farms, valorize nutshell by-products, and help the industry to achieve the zero-waste goal.

In this study, we also observed the negative effect of ethanol (60%, *v*/*v*) on weed growth, especially against the dicot plants (Figure 5). It is intriguing to note that in a previous study using distiller’s dried grains (DDGs), the coproducts leftover from converting corn into ethanol used as livestock feed showed weed-control potential [45]. In that study, DDGs inhibited germination in crabgrass, chickweed, annual rye, and other weeds. However, the precise mechanism of the herbicidal action of DDGs was not identified. We surmised that the residual ethanol in DDGs might be one of the contributing components inhibiting the germination of the weed seeds tested. In this regard, we assumed that the ethanol may have exerted a synergism to the herbicidal activity of the SA when co-applied. It is noteworthy that in a recent study investigating the impact of the solvent composition of herbicidal spray solutions containing choline 2,4-dichlorophenoxyacetate or *N*-hexylcholine 2,4-dichlorophenoxyacetate, the biological activity of the spray solutions was strongly affected by the addition of an organic solvent, such as ethanol, compared to formulations prepared in pure water, and an ethanol-dissolved spray exerted higher phytotoxicity on the target plants [22]. The determination of the precise mechanism of the synergism between SA and ethanol warrants future in-depth investigations.

## 4. Conclusions

Tree-nutshell particles appear to be an effective SA delivery vehicle capable of the controlled release of an SA fumigant. Since tree nuts capture and store an immense amount of carbon over their life-cycles, this new and sustainable use for nutshells not only reduces carbon emissions (carbon sequestration) and costs for waste management but also valorizes tree-nut by-products, thus directly benefitting the tree-nut industry.

SA, a generally-regarded-as-safe (GRAS) molecule possessing intrinsic antifungal potential, exerted promising pre- and post-emergent herbicidal activities against monocot and dicot plants at various growth stages, thus allowing for sustainable weed control in fields. SA dissolved in ethanol (60%, *v*/*v*) might exert synergism with SA after emission from nutshells, which agrees with the results from other investigations performed using 2,4-dichlorophenoxyacetate derivatives (herbicides) dissolved in ethanol (see above). Ethanol-dissolved SA completely disrupted the growth of monocot and dicot plants even without soil coverings, which can help to reduce the consumption of plastics by agricultural and agriculture-related industries.

In summary, if technological advancements are further achieved, we expect that the outcomes of this study will result in effective weed control, significant new markets for tree-nut by-products, the elimination of problem waste, and new revenues for the tree-nut industry.

## Figures and Tables

**Table 1:** Summary of the procedures and methods of SA application.

Methods	Plastic Tarps	Days of Treatments	Solvents	Results
Pre-emergent herbicidal efficacy	Two layers for SA treatment (0.8 and 1.6 M)	7 days * 7 days of SA treatment	DMSO ***	Inhibition of seed germination
Post-emergent herbicidal efficacy (Method 1)	Two layers for SA treatment (0.8 and 1.6 M)	14 days * 7 days of germination and 7 days of SA treatment	DMSO ***	Inhibition of seedling elongation
Post-emergent herbicidal efficacy (Method 2)	One layer for SA treatment (1.6 M)	26 days * 21 days of germination and growth and 5 days of SA treatment	DMSO ***	Wilting of seedlings (death)
Post-emergent herbicidal efficacy (Method 3)	No plastic-covering (0.8 and 1.6 M)	36 days ** 21 days of germination and growth and 3 to 15 days of SA treatment	Ethanol (60%, *v*/*v*)	Wilting of seedlings (death)

* seed sowing date: 12 May 2023; ** seed sowing date: 10 July 2023; *** DMSO, dimethyl sulfoxide.

**Table 2:** Function of weeds as reservoirs of mycotoxigenic fungi that contaminate crops (examples).

Fungi	Mycotoxins	Crops	Weeds	References
*Fusarium graminearum* and *Fusarium asiaticum*	3-acetyldeoxynivalenol (3ADON), 15-acetyldeoxynivalenol (15ADON), and nivalenol (NIV)	Wheat	Gramineous weeds surrounding wheat fields	[40]
*F. graminearum* and *Fusarium avenaceum*	deoxynivalenol (DON), 3-acetyldeoxynivalenol (3ADON), 15-acetyldeoxynivalenol (15ADON), NIV, zearalenone (ZEA), neosolaniol (NEO), enniatin A (ENN A), enniatin A1 (ENN A1), enniatin B (ENN B), enniatin B1 (ENN B1), T-2 toxin (T-2), HT-2 toxin (HT-2), and moniliformin (MON)	Spring wheat	Weeds surrounding spring- wheat fields	[41]
*F. graminearum*, *F. culmorum*, *F. tricinctum*, etc.	Fumonisins, Trichothecenes, and NIV	Maize (seeds)	Major weeds (sixteen): *Chenopodium* *album* L., *Convolvulus arvensis* L., *Echinochloa crus-galli* (L.) *P. Beauvois*, etc. Less common and seldom found weeds (eighteen): *Capsella bursa-pastoris* (L.) *Medicus, Sonchus arvensis* L., *Amaranthus retroflexus* L., etc.	[42]

## Data Availability

The data generated in this study are available in this paper.

## Supplementary Materials

The following supporting information can be downloaded at https://www.mdpi.com/article/10.3390/mps7010001/s1. Figure S1: SA’s antifungal efficacy on tree-nutshells contaminated with environmental fungi. References [6,46] are cited in the Supplementary Materials.
