# Supplementary material for: Valorizing Tree-Nutshell Particles as Delivery Vehicles for a Natural Herbicide
Source: Methods Protoc. 2023 Dec 20;7(1):1. doi: 10.3390/mps7010001 (PMC10892353; doi:10.3390/mps7010001)
Supplement: Supplementary file 1 [file mps-07-00001-s001.zip › mps-2665681-supplementary.pdf]

Supplementary Figure S1

SA Antifungal Efficacy on Tree Nutshells Contaminated with Environmental Fungi

We previously determined that the surfaces of walnut shells were heavily contaminated with environmental microbial pathogens [6]. While walnut shell particles were used as the SA delivery vehicles in this study, we also examined the level of fungal contamination in the nutshells of other tree nuts, namely, almond and pistachios collected from California tree nut farm (Fresno, CA, USA). As shown in **Figure S1a**, nutshells of almond and pistachios also exhibited high level of fungal contamination, confirming tree nut by-products are commonly contaminated with environmental fungi. Noteworthy, tree nut fields in California, USA, are mainly contaminated with *Alternaria* sp., *Aspergillus* sp., *Botrytis* sp., *Botryosphaeria* sp., etc. [46]. While molecular characterization is necessary for confirmation, the fungal contaminants in the nutshells tested appear to include similar kinds of fungi, such as black molds (*Alternaria* sp., *Aspergillus niger*), whitish or dark-gray molds (*Botrytis* sp.) (**Figure S1a).**

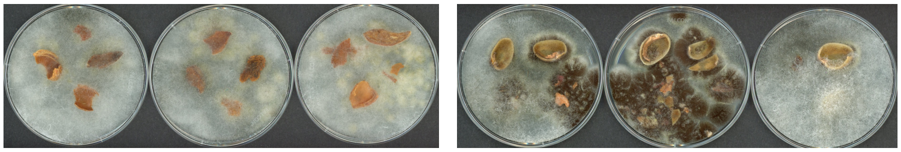

(a)

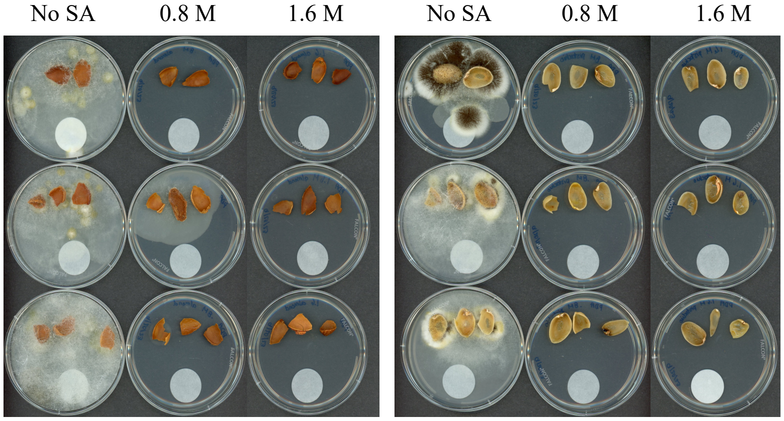

(b)

Figure S1. Antifungal efficacy of SA: (**a**) Fungal contamination in tree nutshells (triplicate plates per assay): (Left) Almond nutshells, (Right) Pistachio nutshells; (**b**) Antifungal efficacy of SA (0.8 and 1.6 M) (triplicates per SA concentration): (Left) Almond nutshells, (Right) Pistachio nutshells.

The antifungal efficacy of SA was also tested against fungi contaminated on the surface of almond and pistachio nutshells. As shown in **Figure S1b**, plates receiving 0.8 to 1.6 M SA almost completely inhibited the growth of fungal contaminants on nutshell particles while the control plate receiving the solvent only (ethanol, 60%, v/v) allowed the growth of fungal contaminants. Thus, as demonstrated in walnut shells [6], the new results with almond and pistachio nutshells further confirm the potent antifungal activity of SA delivered as a fumigant.
